# Linking hydrodynamic disturbances to microeukaryote biogeography: ciliate community shifts reveal freshwater plume and seawater intrusion dynamics

**DOI:** 10.1093/ismeco/ycag055

**Published:** 2026-03-09

**Authors:** Weiwei Liu, Bowen Ye, Zijun Cheng, George B McManus, Gang Li, Linbin Zhou, Dajun Qiu, Jiaxing Liu, Zhixin Ke, Kaizhi Li, Yehui Tan

**Affiliations:** State Key Laboratory of Tropical Oceanography, Guangdong Provincial Key Laboratory of Applied Marine Biology, South China Sea Institute of Oceanology, Chinese Academy of Sciences, Guangzhou 510301, China; State Key Laboratory of Tropical Oceanography, Guangdong Provincial Key Laboratory of Applied Marine Biology, South China Sea Institute of Oceanology, Chinese Academy of Sciences, Guangzhou 510301, China; University of Chinese Academy of Sciences, Beijing 100049, China; State Key Laboratory of Tropical Oceanography, Guangdong Provincial Key Laboratory of Applied Marine Biology, South China Sea Institute of Oceanology, Chinese Academy of Sciences, Guangzhou 510301, China; University of Chinese Academy of Sciences, Beijing 100049, China; Department of Marine Sciences, University of Connecticut, Groton 06340, Connecticut, United States; State Key Laboratory of Tropical Oceanography, Guangdong Provincial Key Laboratory of Applied Marine Biology, South China Sea Institute of Oceanology, Chinese Academy of Sciences, Guangzhou 510301, China; Daya Bay Marine Biology Research Station, South China Sea Institute of Oceanology, Chinese Academy of Sciences, Shenzhen 518121, China; State Key Laboratory of Tropical Oceanography, Guangdong Provincial Key Laboratory of Applied Marine Biology, South China Sea Institute of Oceanology, Chinese Academy of Sciences, Guangzhou 510301, China; State Key Laboratory of Tropical Oceanography, Guangdong Provincial Key Laboratory of Applied Marine Biology, South China Sea Institute of Oceanology, Chinese Academy of Sciences, Guangzhou 510301, China; State Key Laboratory of Tropical Oceanography, Guangdong Provincial Key Laboratory of Applied Marine Biology, South China Sea Institute of Oceanology, Chinese Academy of Sciences, Guangzhou 510301, China; State Key Laboratory of Tropical Oceanography, Guangdong Provincial Key Laboratory of Applied Marine Biology, South China Sea Institute of Oceanology, Chinese Academy of Sciences, Guangzhou 510301, China; State Key Laboratory of Tropical Oceanography, Guangdong Provincial Key Laboratory of Applied Marine Biology, South China Sea Institute of Oceanology, Chinese Academy of Sciences, Guangzhou 510301, China; State Key Laboratory of Tropical Oceanography, Guangdong Provincial Key Laboratory of Applied Marine Biology, South China Sea Institute of Oceanology, Chinese Academy of Sciences, Guangzhou 510301, China; University of Chinese Academy of Sciences, Beijing 100049, China

**Keywords:** ciliate, freshwater plume, seawater intrusion, Pearl River estuary

## Abstract

Estuarine ecosystems are jointly regulated by freshwater plumes and seawater intrusion, yet their impact mechanisms on community dynamics remain insufficiently understood. Here, we investigated the response of ciliate community to freshwater plume-seawater intrusion disturbance in a large subtropical estuary in China. Ciliate distribution exhibited clear turnover along environmental gradients in both community composition and abundance. In summer, community composition showed gradual horizontal and vertical shifts that corresponded with broad environmental gradients generated by strong freshwater plumes. In contrast, in winter, community variation was most pronounced between the inner and middle estuary, reflecting the upstream compression of environmental gradients driven by strong seawater intrusion. These patterns were corroborated by our determinants analyses. Variation partitioning analysis revealed that physical factors explained a substantial proportion of community variation in both seasons, and structural equation modelling further demonstrated that physical factors exerted the strongest total effects on community structure. In addition, species abundances closely followed a log-normal distribution in both seasons, which is consistent with predictions of niche-based community theory. Niche differentiation along environmental gradients shaped by freshwater plumes and seawater intrusion may contribute to this pattern. Overall, our findings reveal a clear linkage between ciliate distribution and freshwater plume-seawater intrusion dynamics, suggesting that ciliate communities can sensitively reflect the spatial–temporal variability of these physical processes.

## Graphical Abstract

**Graphical Abstract:**
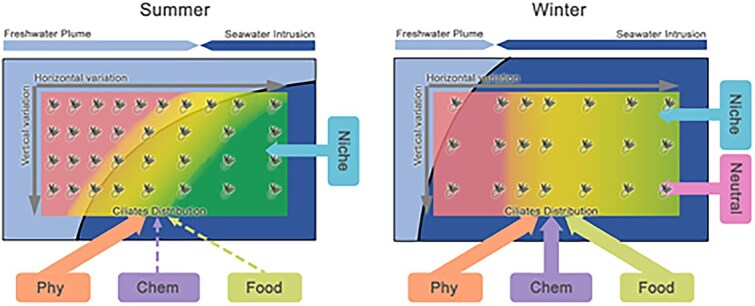

## Introduction

Estuaries are dynamic ecosystems with highly heterogeneous environment. The hydrological interaction of fresh and ocean waters acts as a primary driver of environmental gradients across spatial and temporal scales [1–4]. Spatially, freshwater plume dominates upstream reaches and marine water controls downstream areas, forming sharp environmental transitions along the estuary [5–7]. Temporally, seasonal shifts in river flow (e.g. summer floods) and coastal processes (e.g. storm surges, El Niño events) further amplify this heterogeneity. This complexity, in turn, supports diverse ecological niches that sustain unique assemblages of microplanktons [8–10].

Ciliates are important components of microplanktons and play crucial roles in material cycling and energy flow in aquatic ecosystems [11, 12]. They decompose ingested organic matter into inorganic substances, which can then be reused by producers such as algae, thereby re-entering the material cycle and promoting nutrient regeneration [13, 14]. Meanwhile, as consumers of bacteria and phytoplankton, and prey for mesozooplankton, ciliates serve as a critical link between primary producers and higher trophic levels [15]. Their short generation time, high sensitivity to environmental fluctuations, and strong adaptability to heterogeneous habitats make ciliates ideal bioindicators for characterizing environmental dynamics [16–18]. Some studies have demonstrated that the species diversity, abundance, and community structure of ciliates can be used to assess water quality and discriminate different water masses [19–21].

As the third longest river in China, the Pearl River flows into the South China Sea through the Pearl River Estuary (PRE) with an average runoff of ~5300 m^3^s^−1^ [22–24]. Unlike most estuaries where plume structures are confined to area outside the estuarine mouth, the PRE features diverse plume structures and dynamic hydrodynamic processes throughout the entire estuarine area [25]. Furthermore, freshwater plume intensity is five times higher in summer than in winter, while seawater intrusion extent is three times greater in winter than in summer [26], leading to pronounced seasonal shifts in environmental conditions [27]. Although some investigations on microplankton have been conducted in the PRE [8, 28–31], ciliates have received little attention. Given their high environmental sensitivity, we hypothesize that ciliates will exhibit robust responses to freshwater plume and seawater intrusion, displaying spatiotemporal distribution patterns that are differentially structured and closely aligned with environmental gradients. Specifically, physical factors are expected to exert a stronger influence on ciliate community structure than other environmental variables. Furthermore, the seasonal variation in environmental gradients between summer and winter will drive differences in the assembly mechanisms of ciliate communities, thereby modulating the relative contributions of niche and neutral processes to community assembly.

Here, we investigated the seasonal and spatial variability of the ciliate communities in the PRE, and examined the effects of spatio-temporal environmental dynamics on the communities. Specifically, we addressed the following questions: (i) How do ciliate communities vary along the full environmental gradient across seasonal scales? (ii) Do shifts in ciliate communities reflect the dynamics of freshwater plumes and seawater intrusion? (iii) How do freshwater plume and seawater intrusion influence the biogeographic patterns of ciliates?

## Materials and methods

### Study site and sampling

Samples were collected from a total of 42 and 33 sites across the estuary during summer (16–27 June 2015, wet season) and winter (5–15 December 2015, dry season), respectively (Fig. S1). During the winter cruise, due to poor sea conditions, some sites located in the open sea were inaccessible, resulting in a smaller sampling site size than that in the summer. A total of 222 samples were collected from depths of 0 m, 5 m, 10 m, 20 m, 30 m, 40 m and 50 m (number of water layers for each site was determined based on bottom depth) using a 5 L Niskin Bottle. For ciliate analysis, 1 L samples were immediately fixed with acid Lugol’s iodine solution (2% final concentration) and stored in cold and dark conditions until analysis. For heterotrophic bacteria abundance (HBA), samples were fixed with 2% formaldehyde and quick-frozen in liquid nitrogen. To measure Chl a concentration, 500 ml samples were filtrated sequentially through 20 μm pore-size nylon-net filter, 3 μm polycarbonate filter (Millipore), and 0.7 μm Whatman GF/F filter to determine micro-, nano- and pico-sized Chl a, respectively. The filters were wrapped in aluminum foils and quick-frozen in liquid nitrogen.

We compiled the salinity (S), water temperature (T), pH, dissolved oxygen (DO) data using a YSI 6600 multi-probe sensor (USA). The profiles of photosynthetically active radiation (Kpar) were measured with a PAR sensor (LI-250, LI-COR Inc., USA). In the laboratory, dissolved inorganic nitrogen (DIN) and active silicon (Si) were analyzed using a Seal AutoAnalyzer3 (Bran+Luebbe GmbH, Germany). Dissolved inorganic phosphorus (DIP) was measured using the standard molybdenum blue method. HBA samples were stained with SYBR-Green I (Molecular Probes), and analyzed with a Becton-Dickinson FACSCalibur flow cytometer (Franklin Lakes, NJ, USA). HBA counts were discriminated according to their FL1 (green fluorescence) and side scatter properties. The Chl a filters were extracted with 90% acetone (v/v) in the dark at 4°C for 24 h and measured using a Turner Designs fluorometer 10-AU [27, 29].

### Ciliate identification and enumeration

For identification and enumeration of ciliates, the Lugol’s-fixed subsample was concentrated by settling to 50 ml [32]. One ml of the concentrate was settled in a counting chamber, and all ciliates in the chamber were identified and counted under an inverted microscope at magnifications of 200–400×. Tintinnid ciliates were identified by lorica morphology according to [33–35]. Aloricate ciliates were identified following [36–38]. Uncertain individuals (~2% of all observed individuals) were picked out and identified using protargol impregnation [39]. Moreover, we clarified the trophic trait composition of ciliates by designating species as heterotrophic or mixotrophic type according to the literature [40–42].

### Data analysis

The estuary was divided into three geographic zones based on latitude: the inner zone (>22.35°N), the middle zone (22.05°N–22.35°N), and the outer zone (<22.05°N) (Fig. S1). Horizontal patterns were examined across the three zones. Vertical patterns were analyzed based on water depth within each zone. Samples from depths of 40 m and 50 m were excluded from statistical analyses for vertical pattern due to insufficient sample sizes.

Non-metric multidimensional scaling (NMDS) analysis was conducted to visualize beta diversity. Analysis of similarity (ANOSIM) and permutational multivariate analysis of variance (PERMANOVA) were used to test for significant differences in ciliate communities based on Bray–Curtis dissimilarity. All statistical analyses were performed using the “vegan” package in R [43]. Generalized Additive Models (GAMs) were employed to analyze the horizontal and vertical variation in salinity and ciliate abundance in relation to spatial distance and depth, respectively.

Spearman’s rank coefficients were calculated to assess relationships between ciliate abundance and all environmental factors. Mantel tests were used to explore associations between ciliate community dissimilarity and environmental differences. Detrended correspondence analysis (DCA) was conducted, yielding the maximum gradient lengths of 5.32 in summer and 3.27 in winter. Accordingly, canonical correspondence analysis (CCA) and redundancy analysis (RDA) were performed to explore the relationships between environmental variables and ciliate communities in summer and winter, respectively.

To clarify the influence of freshwater plumes and seawater intrusion on the community, physical factors were used as proxies for these two physical processes. Variation Partitioning Analysis (VPA) was conducted to quantify the relative contributions of physical factors to community variation, and Structural Equation Modeling (SEM) was applied to analyze their direct and indirect effects. Environmental factors were classified into three groups, i.e. physical factors (Phy: referring to S, T, pH, DO, Kpar), chemical factors (Chem: referring to DIN, DIP, Si), and food resource factors (Food: referring to MicroChla, PicoChla, NanoChla and HBA). VPA was conducted using “vegan” package in R, and only the significant variables selected by forward selection were included. SEM analysis was conducted using the “plspm” package in R, treating physical factors (Phy) as exogenous variables, while chemical factors (Chem), food resource factors (Food), and ciliate abundance per sample as endogenous variables. Ciliate community composition (represented by the first NMDS axis) served as the final dependent variable.

Neutral and niche theories predict different species abundance distribution (SAD) models [44]. A log-series distribution, characterized by “few dominants, many rares” arises from random sampling and aligns with neutral theory, which emphasizes random dispersal from a metacommunity [45]. In contrast, a log-normal distribution, marked by “many intermediate-abundance species” reflects stable resource partitioning and corresponds to niche theory, where communities are structured by environmental filtering or resource competition [46]. To evaluate these two models, species abundance data (all species ranked by abundance) were fit to log series and log normal models using the “sads” package in R. Goodness-of-fit was evaluated using the Chi-squared (X2) test and Akaike information criterion.

## Results

### Environmental characterization

All 12 environmental parameters are summarized in Table S1. In general, most environmental parameters were significantly different (T-test, *P* < .01) between summer and winter except for DIN. In summer, the PRE was characterized by low salinity, high Kpar, high nutrient concentrations (e.g. DIP, Si), high HBA abundance and chlorophyll a levels (including MicroChla, NanoChla, PicoChla). In winter, these characteristics were reversed. Detailed description of these environmental parameters can be found in previous studies [27, 29].

### Ciliate community compositions showed seasonal variations

**Figure 1:**
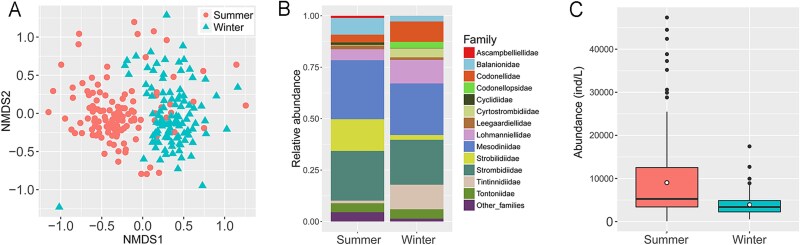
Seasonal comparison of ciliate communities. (A) Non-metric multidimensional scaling (NMDS) ordination of communities from two seasons based on Bray–Curtis dissimilarity. (B) Taxonomic composition and relative abundances of ciliates at family level in two seasons. (C) Comparison of abundance between two seasons, and the circles in the boxes represent the average values.

A total of 251 ciliates species, assigned to 13 classes/subclasses, 49 families and 83 genera, were detected in two seasons. Species richness was higher in summer than in winter (219 vs. 132 species), with 100 species shared between seasons. NMDS revealed clear separation of the communities into two distinct clusters corresponding to summer and winter (Fig. 1A). ANOSIM confirmed that the communities differed significantly between seasons (R = 0.383, *P* = .001, Table 1). In addition, differences of taxonomic composition can be observed between winter and summer. For example, the proportions of Balanionidae (8.1% vs. 2.8%) and Strobilidiidae (15.4% vs. 2.3%) were higher in summer than in winter, whereas Codonellidae (3.7% vs. 9.9%), Cyrtostrombidiidae (0.2% vs. 4.4%), Lohmanniellidae (5.3% vs. 11.5%) and Tintinnidiidae (1.3% vs. 11.9%) were lower in summer than in winter (Fig. 1B). In terms of abundance, average values were significantly higher in summer than in winter (Wilcoxon-test, *P <* .001, Fig. 1C).

**Table 1:** Tests of ciliate community dissimilarity among groupings in horizontal and vertical directions in summer and winter.

		Summer				Winter			
		ANOSIM		PERMANOVA		ANOSIM		PERMANOVA	
Pairwise tests		R	*P*	R2	*P*	R	*P*	R2	*P*
Inner vs. Middle estuary		0.301^**^	<.001	0.077^**^	<.001	0.414^**^	<.001	0.097^**^	<.001
Middle vs. Outer estuary		0.161^**^	.003	0.033^**^	<.001	0.043	.038	0.037^**^	.002
Inner estuary	0 m vs 5 m	−0.111	.704	0.057	.753	−0.092	.905	0.033	.932
Middle estuary	0 m vs 5 m	−0.018	.49	0.092	.075	−0.035	.826	0.026	.808
	5 m vs 10 m	0.493^**^	.009	0.241^**^	.007	−0.088	.731	0.065	.266
	10 m vs 20 m	0.001	.45	0.121	.134	−0.1	.737	0.055	.889
Outer estuary	0 m vs 10 m	0.293^**^	<.001	0.115^**^	<.001	0.078	.068	0.068	.093
	10 m vs 20 m	0.037	.092	0.032	.207	−0.063	.843	0.029	.890
	20 m vs 30 m	−0.175	.946	0.052	.180	0.102	.297	0.091	.390
Global test in horizontal direction		0.283^**^	<.001	0.074^**^	<.001	0.194^**^	<.001	0.082^**^	<.001
Global test in vertical direction		0.144^**^	<.001	0.079^**^	<.001	0.022	.272	0.037^**^	<.001

^^**^^ *P <* .01.

### Horizontal spatial variation of communities and their seasonal dynamics

**Figure 2:**
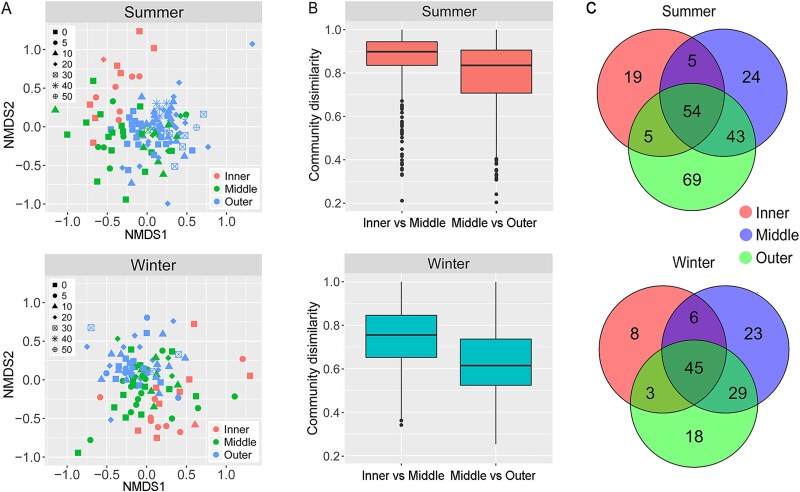
Horizontal variation of ciliate community composition. (A) Non-metric multidimensional scaling (NMDS) ordination of communities in each season based on Bray–Curtis dissimilarity, samples in the three zones coded by color with depths given different shapes. (B) The community dissimilarity between neighboring horizontal zones according to pairwise Bray–Curtis dissimilarity. (C) Venn diagram showing the numbers of unique and shared species in three horizontal zones in summer and winter.

NMDS showed that separation of communities in the three horizontal zones was more distinct than that across different water depths in each season (Fig. 2A), indicating that horizontal variability overrode vertical changes in community structure. Specifically, communities in the inner and outer estuary were separated, while both overlapped with the middle estuary community in both seasons (Fig. 2A). Community dissimilarity between the middle and outer estuary was lower than that between the inner and middle estuary (Fig. 2B. T-test, *P <* .01). This means that the middle estuary community was more similar to that of the outer estuary. Notably the dissimilarities between the middle and outer estuary was lowest in winter. Pairwise ANOSIM indicated that the communities could be significantly distinguished among the three zones in summer, but only between the inner and middle estuary in winter (Table 1). This suggests the horizontal variation was stronger in summer than in winter, and the community variations in winter were mainly confined to the inner and middle estuary transition.

**Figure 3:**
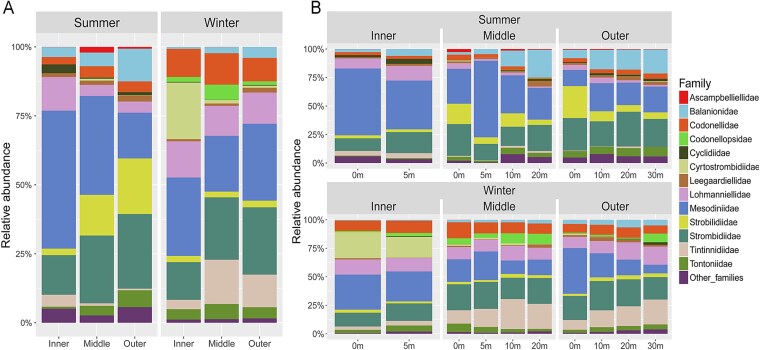
Taxonomic composition at family level and relative abundances of ciliates. (A) In three horizontal zones in two seasons. (B) In different water depths based on three horizontal zones in two seasons.

Venn diagram showed that more species were shared between the outer and middle estuary than either did with the inner estuary across seasons (Fig. 2C). The proportion of shared species between adjacent communities was lower in summer than in winter (inner-middle: 26.9% vs. 38.6%; middle-outer: 44.3% vs. 56.1% Fig. 2C). Family-level taxonomic composition differed among the three zones in both seasons (Fig. 3A), but with the contrasting seasonal trends. For example, the proportions of Lohmanniellidae and Mesodiniidae decreased gradually from inner to outer estuary in summer but remained relatively stable in winter, whereas those of Tintinnidiidae decreased in summer but increased in winter. Generally, in summer, composition patterns showed a clear gradient from the inner to outer estuary. In winter, the middle estuary displayed similar pattern with outer estuary, but remarkably different pattern from the inner estuary, as evidenced by the distribution of Cyrtostrombidiidae and Tintinnidiidae. Continuous spatial sequential further highlighted these patterns. In summer, composition varied sharply across the entire estuarine gradient (Fig. S2). In winter, pronounced changes were only observed at the boundary between the inner and middle estuary (~47–48 km from the innermost site). From middle to outer estuary, the patterns were generally similar.

**Figure 4:**
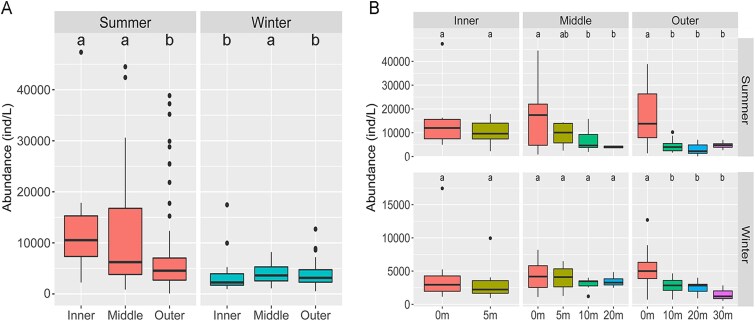
Comparison of ciliate abundances in two seasons. (A) In three horizontal zones. (B) At different depths of three horizontal zones. The different letters above boxes indicate significant differences at the *P <* .05 level according to Wilcoxon test.

Ciliate abundances differed among zones, with significant higher average values in inner and middle estuary in summer and in middle estuary in winter (Wilcoxon-test, *P <* .05, Fig. 4A). The outer estuary consistently exhibited the lowest abundance in both seasons. The highest recorded abundances were 47 350 ind/L at surface of Station Z2 in summer and 17 450 ind/L at Station C3 in winter. Heterotrophic ciliates abundances decreased along the estuarine gradient in summer but followed a unimodal pattern (peaking in middle estuary) in winter (Fig. S3). In contrast, mixotrophic ciliates were consistently more abundant in the inner estuary in both seasons (Fig. S3). Notably, the inner estuary contained several abundance outliers in each season, with values 3–4 times of the zonal average.

### Vertical spatial variation of communities and their seasonal dynamics

Global ANOSIM showed that the communities varied significantly with depth (*P =* .001) in summer but not in winter (*P =* .272) (Table 1). Considering the significant community differences among the three horizontal zones, vertical variation was assessed separately for each zone. Our pairwise ANOSIM results revealed that in summer significant differences between neighboring depths were only found for 5 vs.10 m in the middle estuary and for 0 vs.10 m and 10 vs.20 m in the outer estuary (*P <* .05); no significant differences were found between neighboring depths in winter.

Changes in taxonomic composition with depth were not pronounced in either season, except in the middle and outer estuary in summer (Fig. 3B). For example, in the middle estuary, the proportion of Mesodiniidae at 5 m was twice that at other depths, while in the outer estuary, the proportion of Strobilidiidae at 0 m was nearly four times that at other depths (Fig. 3B).

Ciliate abundance was significantly higher at the surface in the middle and outer estuary in summer as well as the outer estuary in winter (Fig. 4B, Wilcoxon-test, *P <* .05). In terms of the vertical distribution patterns, abundances showed a gradual decreasing trend with depth in the middle estuary and a sharp decline from surface to subsurface layer in the outer estuary in summer. In winter, by contrast, abundance in the middle estuary exhibited almost no vertical variation, while a gradual decreasing trend was observed in the outer estuary (Fig. S4).

### Impacts of environmental factors on communities

Pearson’s correlation analysis showed that environmental variables were more closely correlated with ciliate abundance in summer than in winter (Fig. S5). For example, in summer, most environmental factors except for Kpar were significantly related with ciliate abundance (*P <* .01), whereas in winter only Depth, T, DO, NanoChla and PicoChla showed significant correlations (Fig. S5).

Mantel test showed that the community dissimilarity was significantly correlated with most environmental factors across seasons (Table S2). Salinity was the strongest driver in summer (R = 0.492), whereas silicate (Si) exerted the greatest influence in winter (R = 0.504). T and HBA did not show significant effects in summer but did in winter. CCA and RDA analyses showed that S, pH, T, DO and NanoChla were statistically significantly (*P <* .01) related to community distribution in summer, whereas Kpar, S, Si, MicroChla, NanoChla, and PicoChla were significant in winter (Fig. S6). In both seasons, the first ordination axis generally reflected horizontal variation along the estuary gradient, with S and NanoChla as the most influential variables for summer and winter, respectively.

**Figure 5:**
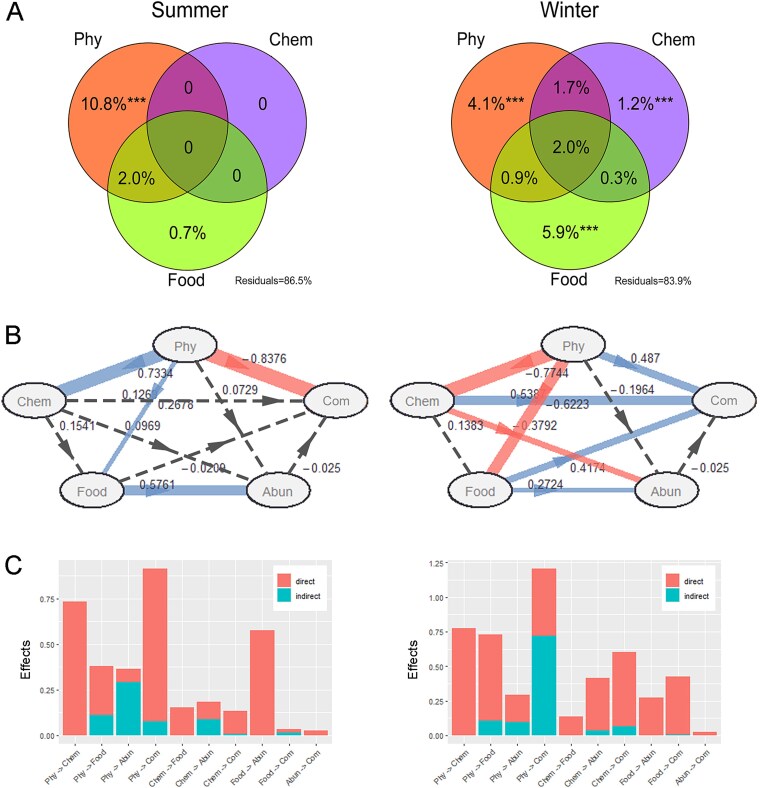
Determinants affecting the ciliate community. (A) Variance partitioning analyses, showing the effects of physical (Phy), chemical (Chem), and food resource (Food) factors on the ciliate community in each season. Values indicate the percentage of variation explained by each fraction, including pure, shared explained and unexplained variability. The variables significantly explaining the variations of ciliate communities after forward selection were used as the subset of Phy, chem, food for each season. ANOVA permutation tests were calculated on the variation explained by each set without the effect of the other. Significance levels are as follows: ^*^P < .05, ^**^P < .01, and ^***^P < .001. (B) Path diagrams showing how physical, chemical and food factors affect the ciliate community composition (Com) and abundance (Abun) in each season. Blue and red solid lines represent significant positive and negative effects, respectively. Grey dashed lines indicate nonsignificant pathways. Path coefficients (numbers on each arrow) indicate the strength of each causal relationship. Models were assessed using goodness of fit (GoF) statistic. The GoFs for each season are 0.51 and 0.47, respectively. (C) Total effects showing the sum of direct and indirect effects for each path.

VPA revealed that the relative contributions of physical, chemical factors, and food resources to the community structure were different between seasons (Fig. 5A). In summer, physical factors explained the largest proportion of the community variation (10.8%), while food factors contributed only 0.7%. No chemical factors showed significant correlation with the community in CCA result and thus they did not explain any variation. In winter, both physical and food factors had strong effects on the community and explained 4.1% and 5.9% of the community variation, respectively, which were significantly higher than chemical factor did (1.2%).

Partial least squares path modelling (PLS-PM) revealed that physical factor exerted significant direct influences on the community in both seasons, with higher effect values in summer than in winter (Fig. 5B). Chemical and food showed significant effects to the community in winter but did not in summer. When combining direct and indirect effects, physical factor was identified as the most dominant driver of community structure in each season (Fig. 5C). For abundance, food exhibited positive direct effects in both seasons. Physical factors did not exert significant direct effects to abundance in either season but indirectly affected it via food (Fig. 5C).

### Species abundance distribution models

To assess potential processes underlying ciliate community assembly in the estuary, we fitted the observed data to log-normal and log-series species abundance distribution (SAD) models (Fig. S7). The observed abundances well matched the log-normal expected lines in summer, but aligned with both log-series and log-normal expectations in winter. The Chi-squared test showed a significant difference between the observed and expected log-series distributions in summer (*P <* .01) but not between the observed and log-normal distributions (*P* > .01). Akaike tests showed the AIC values were significantly lower for log-normal than log-series model (Table 2). These suggested the ciliates assemblages approached the log-normal distributions better than the log-series distribution. In winter, the observed data did not clearly differ from either expected log-normal or log-series distributions in Chi-squared test (*P >* .01, Table 2), and AIC values were comparable between the two models, indicating that the ciliate abundance pattern in winter could be approximately characterized by both log-normal and log-series distributions.

**Table 2:** Results of the species abundance distributions (SAD) model fitting of ciliates in summer and winter in PRE. For each ciliate community, the log-rank abundance curve was compared to model-derived log normal and log-series curves using the X2 and Akaike test. *P* > .01 indicates no significant difference between observed and model expected distributions, and the lower AIC value indicates the closer fit.

	Log-normal			Log-series		
	X2	*P*-value	AIC	X2	*P-*value	AIC
Summer	24.63463	.010307	1889.2	35.15818	.000801	1901.8
Winter	15.49122	.115153	1093.6	14.77346	.254060	1092.4

## Discussion

### Ciliate community displayed significant spatiotemporal variations in PRE

Our study revealed clear seasonal variation in ciliate community. Species richness and abundance were much higher in summer than in winter. Food resource is the key determinant of ciliate abundance [39, 47, 48]. In summer, enhanced freshwater plume transported more nutrients and organic matter into the estuary, boosting ciliate food availability and growth [49–51]. Community composition also differed seasonally. Mixotrophic ciliates such as Mesodiniidae, Strobilidiidae and Strombidiidae were dominant in summer, favored by high nutrient input from freshwater plume. In winter, the proportions of tintinnids such as Condonellidae and Tintinnidiidae were notably higher. These tintinnid species with agglutinated (particle-covered) loricae required large amounts of mineral particles to build their loricae [52]. In winter, seawater intrusion enhanced the vertical mixing of water, which facilitated the suspending of particles [22], and supplied sufficient materials for their loricae building. This was supported by the close relationship between the abundance of these tintinnids and Kpar/Si concentration (Fig. S5). Overall, seasonal environmental fluctuations promoted ecological niche partitioning, driving the difference in ciliate community composition.

Seasonal dynamics of ciliate spatial distribution were rarely examined [53–55]. Our study demonstrated significant seasonal variations in spatial patterns. In summer, the community dissimilarities between adjacent zones were significantly higher and the proportion of shared species was lower than in winter, indicating stronger horizontal variation. ANOSIM showed that the communities could be significantly distinguished among the three zones in summer but only between the inner and middle estuary in winter, suggesting the spatial variation was widespread across the estuary in summer but concentrated in upper estuary in winter. This finding was corroborated by our taxonomy composition patterns, which displayed a clear inner to outer gradient in summer but similar compositions between the middle and outer estuary in winter. Environmental dynamics took great part in the variations of ciliate communities, as revealed by Mantel tests. In summer, freshwater–seawater mixing generated widespread environmental gradients across the estuary [22], driving the gradual community differentiation. In winter, the salinity front moved further upstream due to the enhanced seawater intrusion (Fig. S1). The middle and outer estuary were both dominated by seawater, which likely led to the lower community dissimilarity between these zones. Ciliate abundance peaked in middle estuary in both seasons (Fig. 4A), but heterotrophic and mixotrophic groups exhibited different distribution patterns. Mixotrophic ciliates were consistently abundant in inner estuary across seasons. Impacted by river plume input, the nutrient levels in inner area were elevated [22], which facilitated their autotrophic metabolism [48, 56]. This was supported by the strong positive correlations between abundance of mixotrophic ciliates and concentration of DIN and DIP (Fig. S5). The maximum abundance (40 550 ind/L) was recorded at the surface of Station Z2, attributed to the species *Mesodinium rubrum*. As a typical mixotrophic ciliate, *M. rubrum* can form a high density in eutrophic water especially in estuaries [57], and may even form bloom [58]. The high abundance of *M. rubrum* in inner estuary suggested its potential as an indicator of freshwater plumes [56]. Heterotrophic ciliate abundance was uniform across the estuary in summer but peaked in the middle estuary in winter. In summer, the enhanced nutrient inputs supported widespread food availability along the estuary, leading to similar heterotrophic ciliate abundance. In winter, the freshwater–seawater mixing was concentrated in the middle estuary, where nutrients were efficiently utilized [59]. Consequently, high phytoplankton density occurred there [29, 60], which supported elevated heterotrophic ciliate populations.

Vertical community variation was detected only at intermediate depths in the middle estuary and in surface waters of the outer estuary in summer. Summer salt-wedge circulation generated strong vertical stratification in these regions [27, 61] (Fig. S4), driving the environmental differentiation and ciliate communities divergence. In winter, strong seawater intrusion homogenized environmental properties throughout the water column [22, 50] (Fig. S4), facilitating vertical community exchange and homogeneity. Ciliate abundance patterns mirrored this contrast. In summer, the surface water in the middle and outer estuary had distinctly higher abundances (Fig. S4). Stratification can confine phytoplankton to the upper water with strong irradiance [61, 62], which enhanced food availability and thereby promoted higher ciliate abundance there.

### Contribution of physical factors to distribution of ciliate community

The spatiotemporal distribution pattern of ciliates highlighted the role of freshwater plume and seawater intrusion in shaping community structure. This was corroborated by our environment-correlation analyses. Mantel test and RDA/CCA analyses identified salinity as a key driver of the community variation in the PRE. Ciliate community composition exhibited significant gradient-dependent shift along the salinity continuum (Figs 3, S2, S3). Given that salinity gradients reflected the interaction between fresh and ocean water, this result indicated the effects of freshwater inflow and seawater intrusion on ciliates. Beyond salinity, other physical factors (e.g. temperature, pH, DO, Kpar) also correlated with community variation, indicating that freshwater plumes and seawater intrusion drove community assembly through multiple physical factors.

We quantified the contributions and effects of physical factors on community variation using VPA and SEM analysis to clarify the influence of freshwater plumes and seawater intrusion on ciliate community. VPA result showed that physical factors explained more variation in ciliate distributions than chemical and food factors in both seasons. SEM further revealed that physical factor exerted the strongest total effects on community among all factors. These findings implied the physical processes (specifically freshwater plumes and seawater intrusion) were the primary drivers of ciliate community distribution in the estuary. Consistent with our results, numerous studies have documented the pivotal role of physical processes (e.g. water masses, eddies, and other hydrodynamic features) in regulating ciliate distributions [19]. On the one hand, physical processes can directly alter the spatial distribution of ciliates through advection; on the other hand, they can indirectly influence ciliates by regulating other environmental factors [16, 19]. This was supported by our SEM results, which revealed that physical factor also exerted indirect effects on community via chemical and food factors.

In addition, our results indicated that the effect of physical processes varied seasonally. Both VPA and SEM showed that physical factors had significantly higher explanatory power and effect value on community variation in summer than in winter. Considering that the interaction between freshwater plumes and seawater intrusion was stronger in summer than in winter, i.e. the environmental gradient they generated covered the entire estuary in summer but was confined to the inner and middle estuary in winter [22, 29], it was easy to accept that physical factors had a greater impact on the community in summer. Moreover, VPA revealed that chemical and food factors had extremely low explanatory power in summer, and SEM did not detect significant direct effects of these variables; in contrast, their explanatory power and direct effects increased significantly in winter. The seasonal fluctuations in the availability of food and chemical nutrients likely underpinned this shift [63, 64]. In summer, substantial chemical nutrient inputs from freshwater inflow (Table S1) ensured ample food resources such as phytoplankton, stabilizing predator–prey dynamics between ciliates and their prey and thus weakening food-driven effects on community structure [63]. In winter, reduced nutrient loading decreased food concentrations, making food an important limiting factor for ciliate distribution. This was supported by the high correlation between most food resource variables and ciliate abundance (Fig. S5). Moreover, the shifts in trophic modes can also modulate the effects of food on ciliates. In summer, the proportions of mixotrophic ciliates such as Mesodiniidae, Strobilidiidae and Strombidiidae were high. Their autotrophic metabolism reduced reliance on external food resources and further diminished food-driven community variation. Overall, our study implied that the mechanism through which physical factors influence the community underwent seasonal shift. The intense freshwater–seawater interactions in summer led to strong direct physical effects, whereas weakened freshwater plumes in winter reduced nutrient and food availability, thereby enhancing the indirect effects of physical factors via chemical and food variables.

### Niche differentiation and random dispersal jointly drive ciliate community assembly

An important ecological goal of microbial biogeography is to understand the mechanisms that generate and maintain their diversity [65–68]. Our study offered an opportunity to evaluate whether the neutral or niche-based processes governed the ciliate community assembly in an estuarine ecosystem. SAD analyses revealed that the ciliate communities in both seasons followed log-normal distributions. This aligned with the niche theory, where the abundance of each species in the community is determined by niche differentiation [46]. This agreed with the high explanatory power of physical–chemical for the community variation in VPA, indicating that niche differentiation driven by environmental gradients generated by freshwater plumes and seawater intrusion may structure the ciliate distribution in the PRE.

In addition, the winter community also matched the log-series distribution, indicating that neutral process also took part in structuring ciliate assemblage during this season. This corresponded with the slightly greater explanatory role of spatial factors in winter, and probably reflected the dispersal limitation of species along the estuary. Dispersal limitation of microeukaryotes typically reduces community similarity, as individuals are more likely to colonize nearby habitats [69]. On the one hand, due to the strong seawater intrusion under rising sea levels amplified by global warming, strong environmental gradients were confined to a narrow zone of the PRE, which generated a barrier for ciliate dispersal across the estuary. On the other hand, limited food resources in winter could further restrict dispersal, as consumers may not encounter all available food resources, thereby enhancing dispersal limitation.

### Linking ciliate distribution to freshwater plume–seawater intrusion dynamics

Our results revealed pronounced and consistent associations between ciliate composition, abundance, and distribution and the contrasting hydrodynamic regimes driven by freshwater plumes and seawater intrusion in the estuary. For instance, the relatively high proportion of mixotrophic ciliates in summer corresponded with elevated nutrient inputs delivered by freshwater plumes, whereas the dominance of tintinnids in winter coincided with enhanced particulate suspension associated with seawater intrusion. In summer, the homogeneous spatial variation in ciliate composition aligned with the broad environmental gradients across the estuary. In contrast, the distinct composition changes confined to the upper-middle estuary in winter reflected the upstream compression of environmental gradients under strong seawater intrusion. Vertically, the pronounced differences in ciliate composition and abundance in summer were consistent with stratified water-column conditions induced by freshwater plumes, while the lack of vertical differentiation in winter aligned with vertically mixed water induced by seawater intrusion. Furthermore, the concentration of mixotrophic ciliates in the upper estuary highlighted the influence of the nutrient-rich freshwater inputs. Collectively, these findings underscored the potential of ciliates to reflect the spatial and seasonal variability of freshwater plume–seawater intrusion dynamics.

The stability of estuarine ecosystems relies on the dynamic balance between freshwater plumes and seawater intrusion, which is increasingly influenced by global climate change [70]. Climate-driven sea-level rise intensifies seawater intrusion [71], while extreme events such as floods and droughts associated with El Niño alter estuarine freshwater plume dynamics [72–74]. Such disruptions modify the spatial structure of environmental gradients in estuaries. Given the demonstrated sensitivity of ciliate communities to these hydrodynamic processes, future studies could further evaluate their utility as biological indicators of environmental changes or hydrodynamic anomalies under ongoing climate change.

## Conclusion

**Figure 6:**
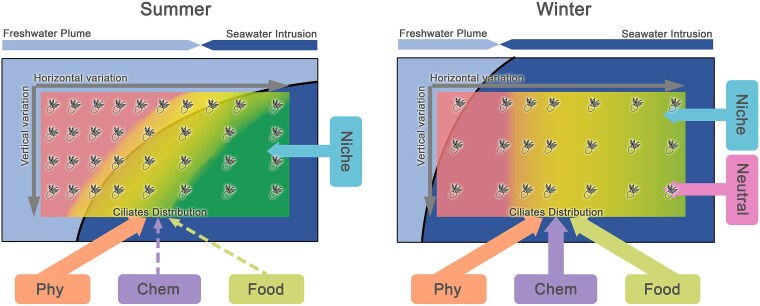
Conceptual models of ciliate distribution patterns, community determinants, and assembly mechanisms effected by freshwater plume (light blue) and seawater intrusion (dark blue). In summer, strong freshwater plume generated broad environmental gradients throughout the estuary, which shapes significant horizontal and vertical differences of ciliate abundance (depicted by ciliate icons density) and composition (depicted by pink-to-green color gradient). Physical factors (Phy) contribute more (with solid arrow) than chemical (chem with dashed arrow), and food resource (food with dashed arrow) to the distribution pattern, and community assembly is dependent on niche-based process. In winter, strong seawater intrusion concentrates the environmental gradient in upstream, where horizontal differences of ciliate abundance and composition can be observed. physical factors (Phy) chemical (chem), and food resource (food) all display strong contribution (with solid arrow) to the distribution pattern, and both niche and neutral processed play roles in the community assembly.

Distribution pattern, determinants and assembling process of the ciliate community were documented in PRE (Fig. 6). In summer, significant horizontal and vertical differences in communities were observed, likely related to the widespread environmental gradients throughout the estuary under strong freshwater plume influence. In winter, by contrast, the community variation was confined horizontally to the transition between inner and middle estuary. This pattern can be attributed to the compression of environmental gradients in upstream driven by intense seawater intrusion. In both seasons, physical factors explained a substantial proportion of community variation, highlighting the pivotal roles of freshwater plumes and seawater intrusion in regulating the ciliate distribution in the estuary. Nevertheless, the mechanism by which physical factors influenced the community shifted seasonally, i.e. they exerted more direct effects in summer but also indirectly affected the community via chemical and food factors in winter. SAD analyses indicated the community assembly was governed by niche-based process (environmental selection) in both seasons, confirming the strong effects of environmental heterogeneity. Overall, this study clarified the mechanism through which freshwater plumes and seawater intrusion drive the seasonal variation of ciliate community in the PRE, and highlighted the potential of ciliates as bioindicators for assessing the ecological effects of physical processes.

## Supplementary Material

Supplementary_material_ycag055

## Data Availability

The data reported in this article are available in Science Data Bank at https://doi.org/10.57760/sciencedb.28664.
