# Supplementary material for: Linking hydrodynamic disturbances to microeukaryote biogeography: ciliate community shifts reveal freshwater plume and seawater intrusion dynamics
Source: ISME Commun. 2026 Mar 9;6(1):ycag055. doi: 10.1093/ismeco/ycag055 (PMC13064646; doi:10.1093/ismeco/ycag055)
Supplement: Supplementary_material_ycag055 [file supplementary_material_ycag055.docx]

**Linking Hydrodynamic Disturbances to Microeukaryote Biogeography: Ciliate Community Shifts Reveal Freshwater Plume and Seawater Intrusion Dynamics**

**Weiwei Liu^1^, Bowen Ye^1, 2^, Zijun Cheng^1, 2^, George B. McManus^3^, Gang Li****^1, 4^, Linbin Zhou^1^, Dajun Qiu^1^, Jiaxing Liu^1^, Zhixin Ke^1^, Kaizhi Li^1^, and Yehui Tan^1, 2^***

**Supplementary material.**

1. **Supplementary Methods**
2. **Supplementary Figures**
3. **Supplementary Table**
4. **Supplementary Methods**

**Horizontal and vertical distributions**

Considering that the influence ranges of freshwater plume and seawater intrusion variated in summer and winter, the spatial patterns of ciliate communities were analyzed based on same geographic groupings in the two seasons in order to compare their responses to seasonal variation of the environmental gradient.

For comparing communities, non-metric multidimensional scaling ordination (NMDS) analyses were conducted and analysis of similarity (ANOSIM) was used to statistically test for significant differences in ciliate communities based on Bray-Curtis dissimilarity. Furthermore, the Bray-Curtis dissimilarities between each pair of samples were analyzed statistically and plotted as a measure of the dissimilarity of ciliate communities. To compare the assemblage composition of spatio-temporal group, the relative abundance of all taxa were plotted at family levels. Wilcoxon test was performed to test the differences in ciliate abundances among horizontal and vertical groups. All statistical analyses were performed with the vegan package in R.

**Relationships between communities and environment**

Spearman’s rank coefficients were calculated to relate ciliate abundance and all environmental factors including the Depth and Distance (from sampling sites to the most inner site of the estuary, C1).

Before multivariate statistical analyses, all environment variables were log (x+1) transformed to improve normality and homoscedasticity. Before the RDA and CCA analysis, a forward selection was conducted to choose significant environmental factors explaining variations in the community data.

We explored the direct and indirect influence of Phy, Chem, and Food factors on ciliate abundance and community composition for each season using the ‘plspm’ (V0.4.7) package in R. This method is known as the partial least squares approach to structural equation modelling and allows for the estimation of complex cause-effect relationship models with latent variables, which was especially suitable for our experimental data with strong environmental gradients.

**Species abundance distribution model analyses**

The mechanisms of community assembly are commonly explained using the neutral and niche theories. These two theories predict different species abundance distribution (SAD) models.

1. **Supplementary Figures**


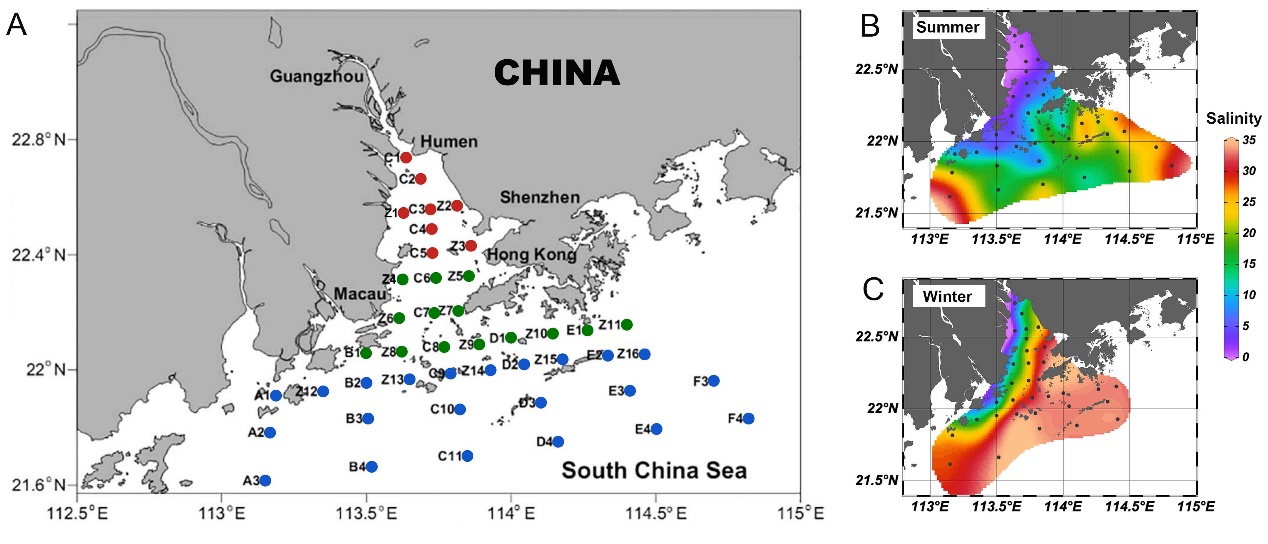


Fig. S1. (A) Map of PRE, with dots showing the sampling sites. To analyses the spatial variations of ciliates, the estuary was divided into three geographic zones, i.e. inner zone (red), middle zone (green), and outer zone (blue). (B, C) Distribution of salinity on surface water of PRE in summer (B) and winter (C).


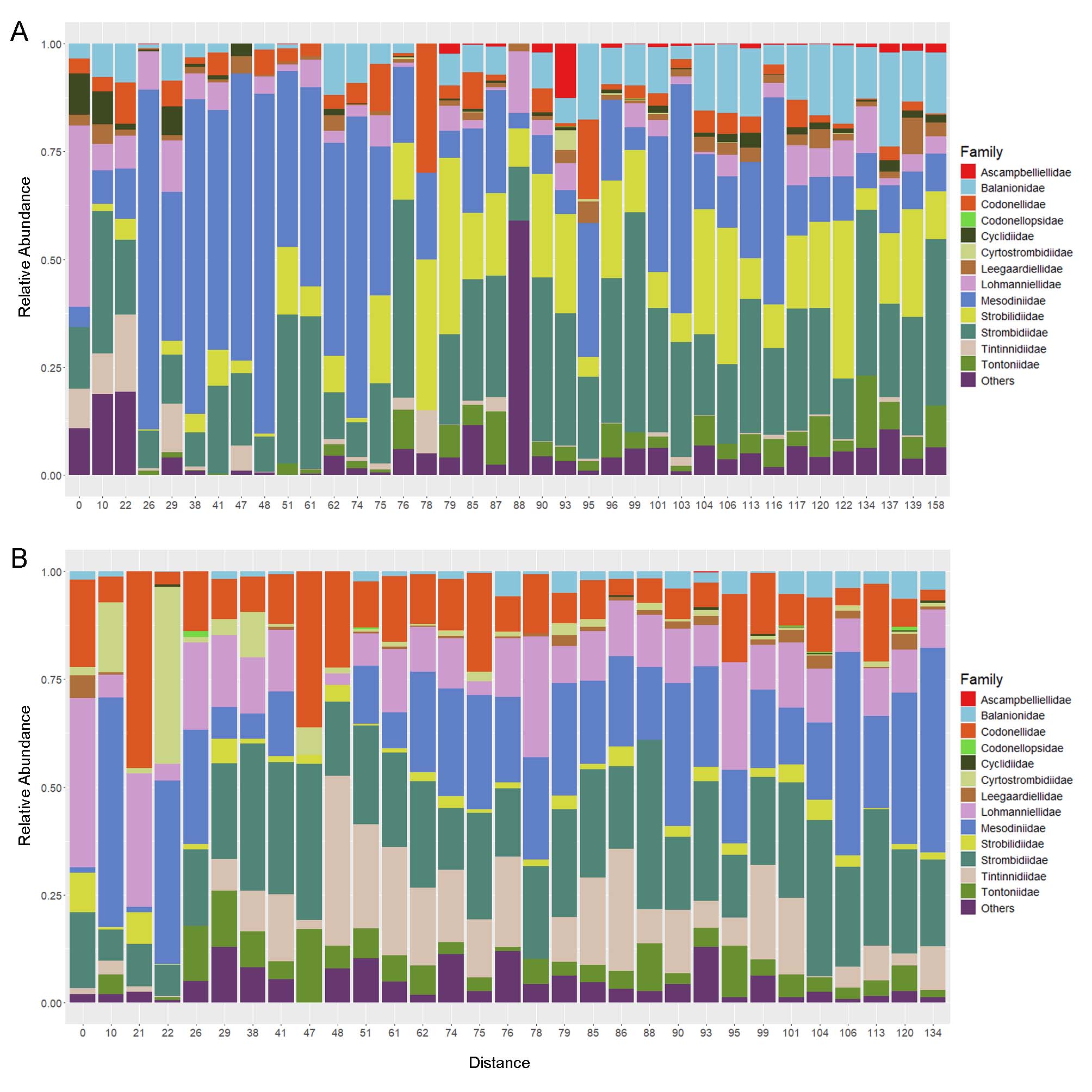


Fig. S2. Taxonomic compositions at family level of ciliates in terms of relative abundances in surface sample along the estuary (distances from sample sites to innermost site, km) in summer (A) and winter (B).


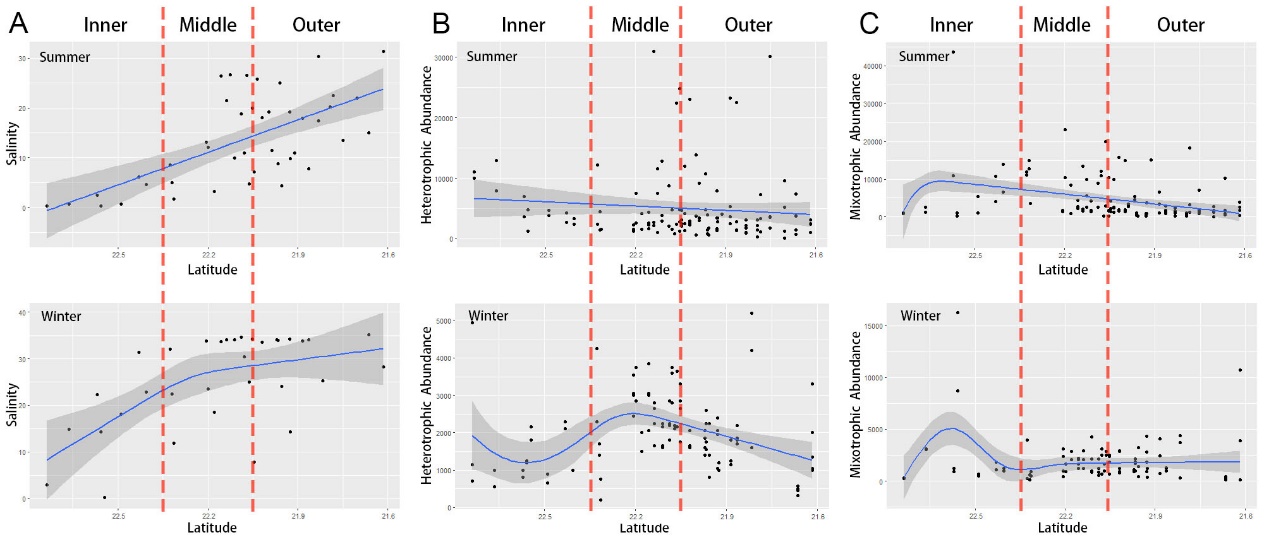


Fig. S3. Horizontal distributions of salinity (A), heterotrophic (B) and mixotrophic (C) ciliate abundances along the estuary in each season. Black points indicate observed data for each sample. Blue lines represent generalized additive model (GAM) fits.


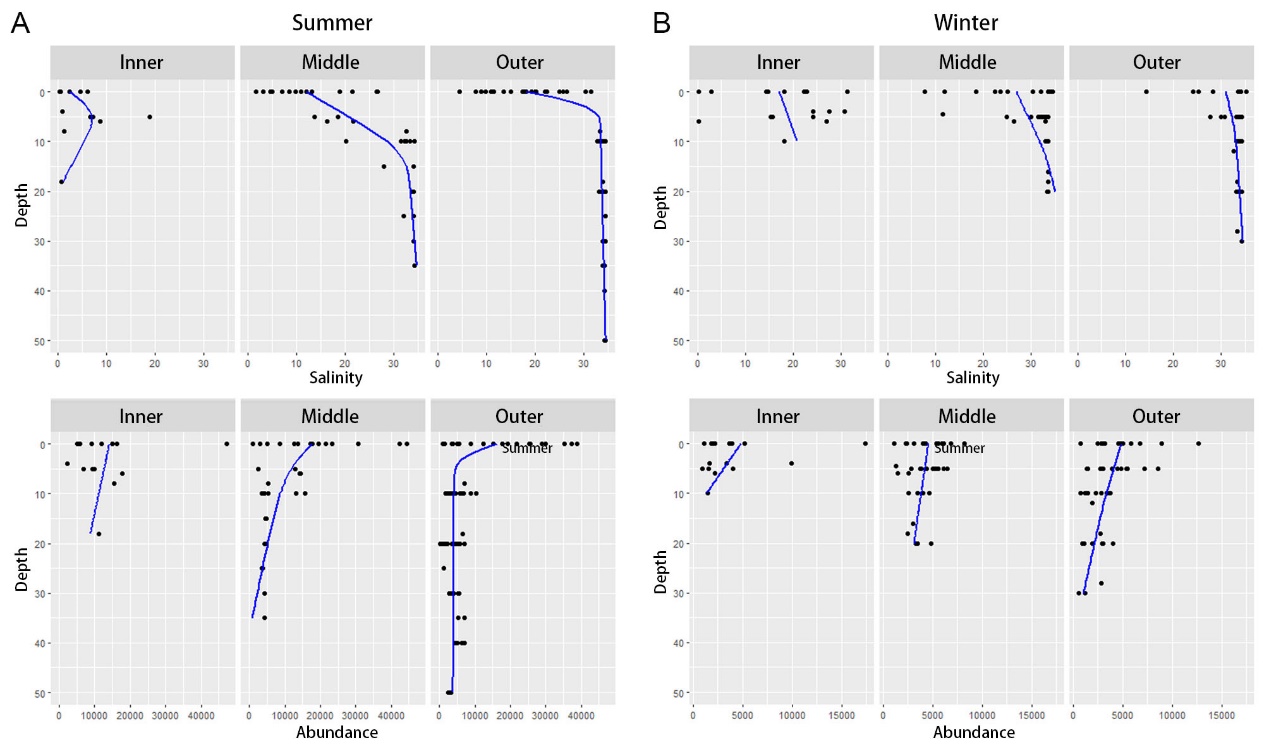


Fig. S4. Vertical distributions of salinity and ciliate abundances along the water depth in inner, middle and outer part of PRE in summer (A) and winter (B). Black points indicate observed data for each sample. Blue lines represent generalized additive model (GAM) fits.


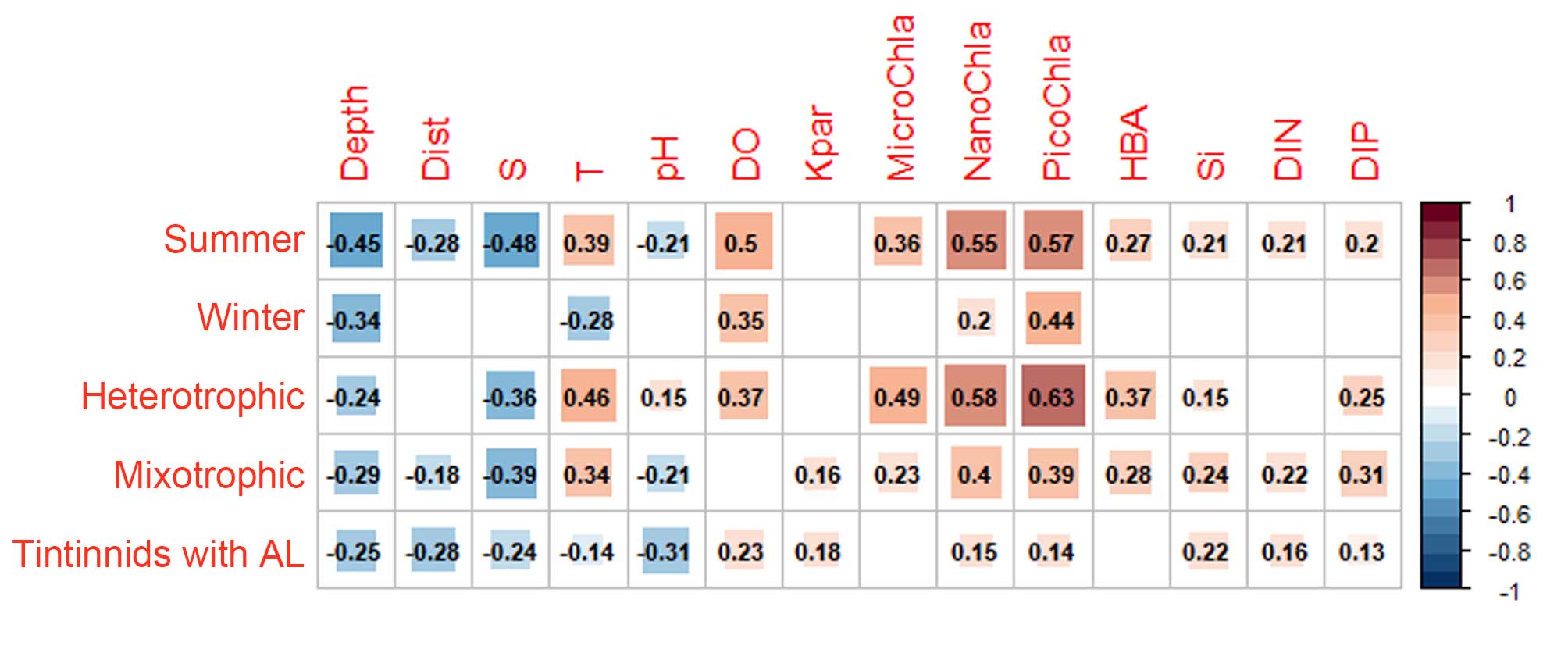


Fig S5. Pearson’s correlation analyses between environmental variables and ciliate abundance. Depth: water depth; Dist: distances from sample sites to innermost site; DIN: dissolved inorganic nitrogen; DIP: dissolved inorganic phosphorus; HBA: heterotrophic bacteria abundance; Heterotrophic: abundance of heterotrophic ciliate; Kpar: light extinction coefficient; MicroChla: microplankton chlorophyll a (>20 μm); Mixotrophic: abundance of mixotrophic ciliate; NanoChla: nanoplankton chlorophyll a (3-20 μm); PicoChla: picoplankton chlorophyll a (<3 μm); S: salinity; Si: active silicon; Summer: ciliate abundance in summer; T: temperature; Tintinnids with AL: abundance of tintinnid ciliate with agglutinated loricae; Winter: ciliate abundance in winter.


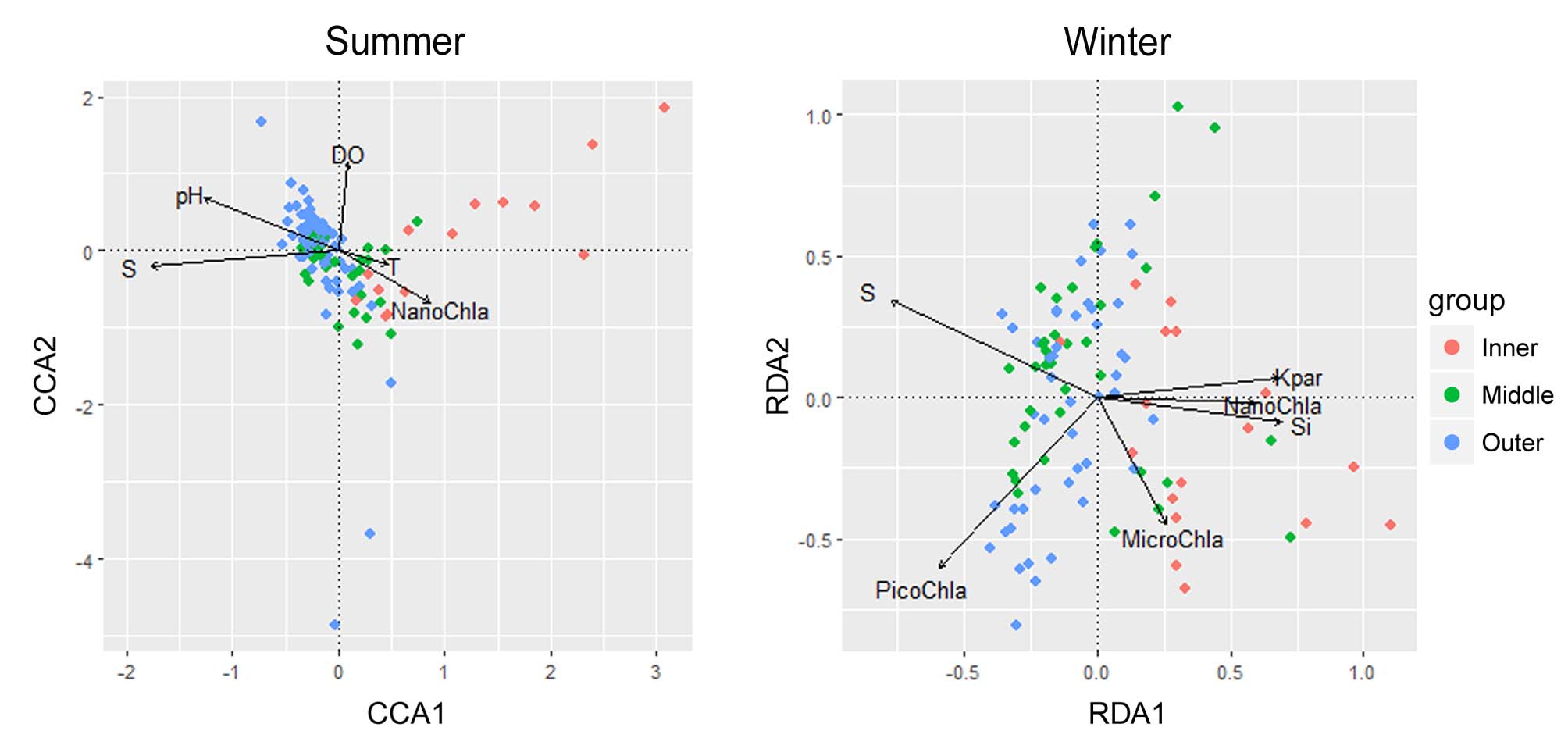


Fig. S6. CCA and RDA ordination showing the ciliate communities in relation to the environmental variables in summer and winter, respectively. Only statistically significant environmental variables (chosen by forward selection) are shown.; DO: dissolved oxygen; Kpar: light extinction coefficient; MicroChla: microplankton chlorophyll a (>20 μm); NanoChla: nanoplankton chlorophyll a (3-20 μm); PicoChla: picoplankton chlorophyll a (<3 μm); S: salinity; Si: active silicon; T: temperature.


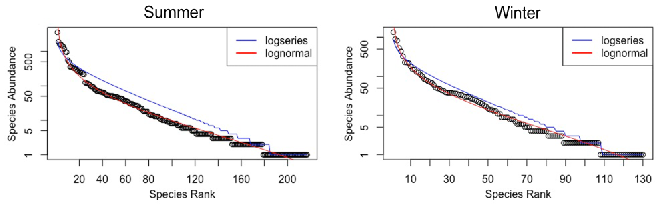


Fig. S7. Log-rank abundance curves for species abundance distribution of ciliates in Summer and Winter. Actual observed abundances are shown as circles; lines are the expected abundances from two distribution models: log-series (blue line) and log-normal (red line)

1. **Supplementary Table**

Table S1. Summary of environmental parameters in summer and winter.

| Parameter | Summer (n=122) | | | | Winter (n=100) | | | | *p-*value  (T-test) |
| --- | --- | --- | --- | --- | --- | --- | --- | --- | --- |
|  | Max | Min | Mean | SD | Max | Min | Mean | SD |  |
| S (PSU) | 34.35 | 0.337 | 24.349 | 11.853 | 35.178 | 0.176 | 29.045 | 8.053 | <0.001 |
| T (°C) | 31.667 | 21.03 | 26.276 | 3.071 | 22.77 | 17.738 | 20.469 | 1.091 | <0.001 |
| pH | 8.73 | 7.02 | 7.913 | 0.367 | 7.95 | 6.332 | 7.649 | 0.271 | <0.001 |
| DO (mg L-1) | 11.547 | 0.435 | 5.825 | 1.996 | 8.325 | 5.613 | 7.131 | 0.422 | <0.001 |
| Kpar (m-1) | 2.252 | 0.163 | 0.568 | 0.494 | 1.03 | 0.171 | 0.402 | 0.18 | 0.007 |
| DIN (umol kg-1) | 133.639 | 0.815 | 34.649 | 37.933 | 113.093 | 1.68 | 26.399 | 30.063 | 0.072 |
| DIP (umol kg-1) | 53.871 | 0.001 | 14.518 | 16.659 | 1.741 | 0.174 | 0.453 | 0.309 | <0.001 |
| Si (umol kg-1) | 219.36 | 2.137 | 65.098 | 62.641 | 193.929 | 4.214 | 43.865 | 42.969 | 0.0032 |
| HBA (cells ml-1) | 12134 | 613 | 2013.32 | 1410.508 | 1246.8 | 281.4 | 793.474 | 236.641 | <0.001 |
| MicroChla (ugL-1) | 5.582 | 0.033 | 0.887 | 1.131 | 1.154 | 0.079 | 0.289 | 0.142 | <0.001 |
| NanoChla (ugL-1) | 12.344 | 0.06 | 1.922 | 2.268 | 2.831 | 0.179 | 0.821 | 0.423 | <0.001 |
| PicoChla (ugL-1) | 6.7 | 0.051 | 1.083 | 1.417 | 1.261 | 0.112 | 0.491 | 0.253 | <0.001 |

DO: dissolved oxygen; DIN: dissolved inorganic nitrogen; DIP: dissolved inorganic phosphorus; HBA: heterotrophic bacteria abundance; Kpar: light extinction coefficient; MicroChla: microplankton chlorophyll a (>20 μm); NanoChla: nanoplankton chlorophyll a (3-20 μm); PicoChla: picoplankton chlorophyll a (<3 μm); S: salinity; Si: active silicon; T: temperature. T-test was performed to indicate the difference significances of each parameter between summer and winter

Table S2. Mantel tests for the correlation between community similarity and environmental variables using Pearson’s coefficient.

| Parameter | | Global | S | T | pH | DO | Kpar | DIN | DIP | Si | HBA | MicroChla | NanoChla | PicoChla |
| --- | --- | --- | --- | --- | --- | --- | --- | --- | --- | --- | --- | --- | --- | --- |
| Summer | R | 0.524*** | 0.492 *** | 0.067 * | 0.397 *** | 0.177 *** | 0.376 *** | 0.351 *** | 0.351 *** | 0.393 *** | 0.078 | 0.097 * | 0.3 *** | 0.143 ** |
|  | *p* | 0.0001 | 0.0001 | 0.0131 | 0.0001 | 0.0002 | 0.0001 | 0.0001 | 0.0001 | 0.0001 | 0.1056 | 0.0441 | 0.0001 | 0.0086 |
| Winter | R | 0.581*** | 0.488 *** | 0.463 *** | 0.262 *** | 0.382 *** | 0.399 *** | 0.474 *** | 0.275 *** | 0.504 *** | 0.204 *** | 0.192 * | 0.392 *** | 0.137 ** |
|  | *p* | 0.0001 | 0.0001 | 0.0001 | 0.0002 | 0.0001 | 0.0001 | 0.0001 | 0.0003 | 0.0001 | 0.0003 | 0.0102 | 0.0001 | 0.0083 |

DO: dissolved oxygen; DIN: dissolved inorganic nitrogen; DIP: dissolved inorganic phosphorus; HBA: heterotrophic bacteria abundance; Kpar: light extinction coefficient; MicroChla: microplankton chlorophyll a (>20 μm); NanoChla: nanoplankton chlorophyll a (3-20 μm); PicoChla: picoplankton chlorophyll a (<3 μm); S: salinity; Si: active silicon; T: temperature. ****p*<0.001; **0.001<*p*<0.01; *0.01<*p*<0.05
